# Hemolytic uremic syndrome following *Hemiscorpius lepturus* (scorpion) sting

**DOI:** 10.4103/0971-4065.45293

**Published:** 2008-10

**Authors:** E. Valavi, M. J. Alemzadeh Ansari

**Affiliations:** Department of Nephrology, Abuzar Pediatric Hospital, Ahwaz, Iran; 1 Faculty of Medicine, Jundishapour University of Medical Sciences, Ahwaz, Iran

**Keywords:** Hemolytic uremic syndrome, microangiopathic hemolytic anemia, scorpion sting

## Abstract

Scorpion envenomations are a public health problem in many countries. Scorpions are second only to snakes in causing human fatalities from envenomation. Species of scorpions capable of inflicting fatal stings are living in North and South Africa, the Middle East, India, America, Trinidad, and Tobago. *Hemiscorpius lepturus* (from the Hemiscorpiidae family) is the most medically important scorpion in Iran which accounts for 92% of all hospitalized scorpion sting cases. The venom from *H. lepturus* is primarily a cytotoxic agent and has hemolytic, nephrotoxic, and to some extent, hepatotoxic activities. We found a combination of microangiopatic hemolytic anemia, thrombocytopenia, and acute renal failure in a seven year-old female child who was referred to us with a 12 h history of bloody urine following a *H. lepturus* sting. Her blood smear showed fragmented erythrocytes and burr cells, leading us to a diagnosis of hemolytic uremic syndrome (HUS). This report highlights the importance of acceptable prophylaxis and therapeutic protocols for HUS in these patients.

## Introduction

Hemolytic uremic syndrome (HUS) is characterized by thrombocytopenia, hemolytic anemia, and acute renal impairment, which has been classified into diarrhea-related and nondiarrhea related types in some reports. About 90% of diarrhea-related HUS occurs in early childhood and presents with bloody diarrhea. Etiology of nondiarrhea-related HUS could be disorders of complement regulation, collagen vascular disease, HIV infection, malignancy, and drugs.^1^ Disseminated intravascular coagulation and microangiopathic hemolytic anemia are well known complications following scorpion stings.^2^ However, we found hemolytic uremic syndrome in our case after a sting of the *Hemiscorpius lepturus* scorpion.

## Case Report

A seven year-old female child presented in July 2007 with a 12 h history of bloody urine, vomiting, agitation, and restlessness following a scorpion sting; health care services confirmed that the culprit species was *Hemiscorpius lepturus*. Examination revealed that she had a tender and erythematic area with local edema on the right leg (4 × 6 cm); the patient was conscious. Her temperature was 39.6°C, pulse was 125/min; blood pressure was normal (100/60 mmHg). At the time of admission, hematology revealed hemoglobin = 10.2 g/dL, white blood cells = 24,000/mm^3^, and platelets = 175,000/mm^3^; urinary analysis showed 3+ hemoglobinuria and microscopic hematuria without proteinuria. Serum creatinine (SCr) and blood urea nitrogen (BUN) levels were normal (0.4 and 25 mg/dL, respectively); other blood chemistry results were unremarkable. Coomb's test was negative and the G6PD level and peripheral blood smear were normal. At this time, the patient received polyvalent antivenom, cefazoline, and 20 meq/L sodium bicarbonate in dextrose water.

During the first week, SCr and BUN levels gradually rose (8.6 and 100 mg/dL, respectively), but the hemoglobin level and platelet count dropped to 8 g/dL and 47,000/mm^3^ respectively. A blood smear showed fragmented erythrocytes and burr cells; total serum protein level was 5.9 g/dL, albumin level was 3.3 g/dL, calcium level 9.2 mg/dL, phosphorus level 6.2 mg/dL, and lactate dehydrogenase 16358 IU/L. Urinary analysis showed 3+ proteinuria, 3+ hemoglobinuria, and erythrocyte casts. Prothrombin time and partial thromboplastin time were normal (12 and 32 seconds, respectively). A sonogram of the urinary tract was normal. Bone marrow aspiration revealed essential hypercellular marrow with increased megakaryocytes, erythroid hyperplasia, and a shift to the left in myelopoiesis. She received packed cell, fresh, frozen plasma and platelets several times. Also, hydrocortisone (10 mg/kg/day) was added and hemodialysis was performed. In the second week, platelets rose to normal levels (248,000/mm^3^) but the lactate dehydrogenase level dropped to 1430 IU/L. We did not do renal biopsy but the combination of microangiopatic hemolytic anemia, thrombocytopenia, and acute renal failure led us to diagnose hemolytic uremic syndrome in this child. After one month, she was discharged with a SCr level of 6.7 mg/dl on an every-other-day dialysis program.

## Discussion

The term “hemolytic uremic syndrome” was coined by Conrad von Gasser *et al*. in 1955 to describe a devastating illness consisting of acute renal failure accompanied by nonimmune hemolytic anemia and thrombocytopenia.^13^

HUS is part of the disease cluster called thrombotic microangiopathies and is characterized by thrombocytopenia, microangiopathic hemolytic anemia, and acute renal impairment. It is the most common cause of acute renal failure in children.^4^ Thrombotic microangiopathies are characterized by fragmentation of erythrocytes and extremely elevated serum levels of lactate dehydrogenase. The severity of these abnormalities reflects the extent of the microvascular aggregation of platelets. Helmet cells, burr cells, and fragmented erythrocytes are probably produced as blood flows through turbulent areas of the microcirculation that are partially occluded by platelet aggregates—this process causes microangiopathic hemolytic anemia. Serum lactate dehydrogenase is largely derived from ischemic or necrotic tissue cells rather than from lysed red cells.^5^,^6^ HUS and thrombotic thrombocytopenic purpura represent different ends of what is probably the same disease continuum. Endothelial cell injury appears to be the primary event in the pathogenesis of these disorders. The endothelial damage triggers a cascade of events that result in microvascular lesions with platelet-fibrin hyaline microthrombi that occlude arterioles and capillaries, which cause its complications such as acute renal failure, stroke, bowel necrosis perforation, and intussusception. Platelet aggregation results in a consumptive thrombocytopenia; epithelial damage may result from toxins released by bacteria or viruses.^6^

HUS is subdivided into two forms, depending on whether the patient has had diarrhea. Acute enteritis with diarrhea caused by *E. coli* O157: H7 precedes approximately 90% HUS cases. Nondiarrhea-related HUS, which accounts for 10% of HUS cases, is sometimes referred to as atypical HUS. Causes of thrombotic microangiopathies vary widely and they have recently been divided into two groups, based on understanding and not fully understanding causes. Circumstances where the etiology is well understood include induction by an infection (Shiga and verocytotoxin-producing bacteria or *Streptococcus pneumoniae*), disorders of complement regulation (genetic or acquired disorders of complement regulation), ADAMTS13 (a disintegrin and metalloprotease with thrombospondin type 1 repeats) abnormalities (ADAMTS13 deficiency secondary to mutations or autoantibodies against ADAMTS13), defective cobalamine metabolism, and quinine-induced TMA. Diseases associated with thrombotic microangiopathies where the etiology is not so well defined include HIV, malignancy, drugs (including cytotoxics, immunosuppressant, oral contraceptives, and antiplatelet agents), pregnancy, systemic lupus erythematosus, and antiphospholipid antibody syndrome.^1^,^7^ Two recently published reports describe HUS following scorpion stings.^8^,^9^

After a *H. lepturus* sting, various degrees of local toxicity are observed in 80% of the cases which include macular erythema, purpuric changes, bulla, necrosis, and ulcers.^10^ General symptoms include dry mouth, dizziness, nausea, vomiting, fever, and other symptoms associated with stimulation of the sympathetic and parasympathetic systems. Patients who develop severe toxicities are more likely to have fever, confusion, convulsion, hemoglobinuria, and reduction in hemoglobin level below 10 g/dL. Renal toxicity which is one of the serious systemic effects, can progress to severe renal and cardio-respiratory failure if not treated early by administration of the polyvalent antivenom, and up to 20% of these cases may need dialysis. Hemolytic anemia and renal failure have higher probability of incidence in children younger than 10 years of age. The mortality rate due to *H. lepturus* stings has reached > 8% in the past several years, particularly among pediatric patients. Histological findings support the hypothesis that the venom from this scorpion is primarily a cytotoxic one, as shown by the dermonecrotic effects as well as the widespread damage to all of the nephron segments.^11^,^12^ Disseminated intravascular coagulation and vascular injuries are well known complications following scorpion stings, and there are several reports of thrombotic and microangiopathies events (*i.e*., stroke and multiple cerebral and cerebellar infarctions) that explain vascular injuries following envenomation.^2^,^13^

We conclude that microangiopathic hemolytic anemia, hemolytic uremic syndrome, and thrombotic complications are due to cytotoxic effects and vascular injuries following scorpion envenomation. Hence, it is very important to establish HUS prophylaxis and acceptable therapeutic protocols

## References

[1] Jokiranta TS, Zipfel PF, Fremeaux-Bacchi V, Taylor CM, Goodship TJ, Noris M (2007). Where next with atypical hemolytic uremic syndrome?. Mol Immunol.

[2] Gadwalker SR, Bushman S, Pramond K, Gouda C, Kumar PM (2006). Bilateral cerebellar infarction: A rare complication of scorpion sting. J Assoc Physicians India.

[3] Gasser VC, Gautier E, Steck A, Siebenmann RE, Oechslin R (1955). Hämolytisch-ürämische Syndrome: Bilaterale Nierenrindennekrosen bei akuten erworbenen hämolytischen Anämien. Schweiz Med Wochenschr.

[4] Elliott EJ, Robins-Browne M (2005). Hemolytic uremic syndrome. Curr Probl Pediatr Adolesc Health Care.

[5] Moake JL (2002). Thrombotic Microangiopathies. N Engl J Med.

[6] Franchini M, Zaffanello M, Veneri D (2006). Advances in the pathogenesis, diagnosis and treatment of thrombotic thrombocytopenic purpura and hemolytic uremic syndrome. Thromb Res.

[7] Kavanagh D, Richards A, Noris M, Hauhart R, Liszewski MK, Karpman D (2008). Characterization of mutations in complement factor I (CFI) associated with hemolytic uremic syndrome. Mol Immunol.

[8] Mocan H, Mocan MZ, Kayanar K (1998). Hemolytic uremic syndrome following a scorpion sting. Nephrol Dial Transplant.

[9] Bahloul M, Ben Hamida M, Belhoul W, Ksibi H, Kallel H, Ben Hamida C (2004). Hemolytic-uremic syndrome secondary to scorpion envenomation (apropos of 2 cases). Nephroloqie.

[10] Radmanesh M (1998). Cutaneous manifestations of the Hemiscorpius lepturus sting: A clinical study. Int J Dermatol.

[11] Pipelzadeh MH, Jalali A, Taraz M, Pourabbas R, Zaremirakabadi A (2007). An epidemiological and a clinical study on scorpionism by the Iranian scorpion *Hemiscorpius lepturus*. Toxicon.

[12] Pipelzadeh MH, Dezfulian AR, Jalali MT, Mansouri AK (2006). In vitro and in vivo studies on some toxic effects of the venom from *Hemiscorpious lepturus* scorpion. Toxicon.

[13] el Nasr MS, Abdel Rahman M, Shoukry NA, Ahmed MK, Eid SM, Fawzi AF (1992). The effect of scorpion envenomation on the different organs of albino mice. J Egypt Soc Parasitol.

